# Comparative antibacterial efficacy of MTA, amniotic membrane, and hyaluronic acid in pulp therapy for primary teeth

**DOI:** 10.1186/s12903-025-07038-3

**Published:** 2025-11-05

**Authors:** Eman Ibrahim, Dina Hamdy, Nagwa Khattab

**Affiliations:** Pediatric Dentistry and Dental Public Health, Faculty of Dentistry, Ain Shams University, Cairo, Egypt

**Keywords:** Amniotic membrane, Capping material, Formcresol, Inhibition zone, Primary teeth, Pulpotomy

## Abstract

**Background:**

Pulp therapy is a widely used treatment for cariously exposed asymptomatic primary teeth. Formocresol (FC) has traditionally been considered the standard treatment for pulpotomy, but its safety has raised concerns in recent years. Consequently, various alternative materials were explored and proposed.

**Aim:**

The purpose of this study is to compare the anti-bacterial effects of three pulp capping materials against *Enterococcus faecalis*,* Streptococcus mutans*,* and Lactobacillus acidophilus*.

**Methods:**

The antibacterial effect of the three pulp capping materials was evaluated using the agar diffusion method, Group I; MTA, Group II: amniotic Membrane, Group III: hyaluronic acid against *Streptococcus mutans*,* Enterococcus faecalis*,* and Lactobacillus acidophilus.*

**Results:**

A statistically significant difference was observed among the tested groups. MTA exhibited the largest inhibition zone (18.25 ± 1.71 mm) against *E.faecalis*, followed by the amniotic membrane (11.75 ± 3.86 mm), while hyaluronic acid showed no antibacterial activity.Similarly, a significant difference was observed in inhibition zones among the tested groups against *Streptococcus mutans* (*p* < 0.001), where MTA showed the highest inhibitory effect (13.50 ± 1.2 mm), followed by hyaluronic acid (12.00 ± 0.82 mm), whereas the amniotic membrane exhibited no antibacterial effect. *Lactobacillus acidophilus* was not affected by any of the tested materials in terms of antibacterial activity.

**Conclusions:**

MTA and the amniotic membrane demonstrated effectiveness against *Enterococcus faecalis*, while hyaluronic acid showed no antibacterial activity against this strain. Both MTA and HA exert antibacterial effects against Streptococcus mutans, whereas the amniotic membrane had no effect. The tested materials were ineffective against Lactobacillus acidophilus, which showed resistance. Overall, MTA displayed the strongest antibacterial effect among the tested groups.

**Supplementary Information:**

The online version contains supplementary material available at 10.1186/s12903-025-07038-3.

## Background

Vital pulp therapy is intended to preserve and maintain the health of the dental pulp in teeth that have been injured, aims to protect and sustain pulp health in teeth exposed to injury, decay or unsuccessful restorative procedures. The Mineral trioxide aggregate is among the materials with strong regenerative potential. It is both biocompatible and bioinductive, and has been studied for use in pulp therapy [1–3].

Mineral trioxide aggregate (MTA) is a calcium silicate-based cement mainly consisting of tricalcium silicate (Ca₃SiO₅), dicalcium silicate (Ca₂SiO₄), tricalcium aluminate (Ca₃Al₂O₆), calcium sulfate (CaSO₄), bismuth compounds, and hydrophilic particles that set upon exposure to moisture. MTA was first offered as a material for perforation sealing and root-end repair. Elevated pH confers antibacterial activity. MTA is a biocompatible cement. Nevertheless, it has some acknowledged downsides, like an increased price, a prolonged setting time, and the possibility of discoloration [4].

The amniotic membrane matrix is abundant in growth factors (GFs), For instance, the basic fibroblast growth factor (b-FGF), keratinocyte growth factor (KGF), epidermal growth factor (EGF) and transforming growth factor-beta (TGF-β), all of which facilitate tissue restoration. These growth factors replicate the natural growth environment, creating an optimal setting for healing [5]. It functions as a structural support that promotes differentiation, proliferation and tissue regeneration, thanks to the presence of fibronectin, laminins, collagen types I, III, IV, V, and VI, proteoglycans, elastin and hyaluronic acid in its stromal layer. This makes it an ideal candidate for use as An inherent framework in tissue engineering [5, 6].

Additionally, the amniotic membrane releases essential nutrients, facilitates cell migration, adhesion, and differentiation, and helps modulate the semi-allogeneic immune response toward the fetus. Moreover, it possesses biological, antimicrobial (such as beta-defensins), and anti-inflammatory properties comparable to those of cortisone and steroids. The anti-fibrotic, anti-scarring, anti-angiogenic, and pain-relieving characteristics make it a distinctive treatment for wound healing and a favorable environment for enhancing the survival and growth of mesenchymal progenitor cells [7–9]. Research has demonstrated that mesenchymal and epithelial stromal cells derived from the human amniotic membrane produce antimicrobial compounds that contribute to the innate immune response. Among these, α and β defensins display antibacterial, antiviral, and antifungal properties. In addition, proteins like secretory leukocyte protease inhibitor (SLPI) and elafin, which contain Whey Acidic Protein (WAP), are recognized for their antimicrobial and anti-protease functions [10–15].

Recently, hyaluronic acid (HA) has garnered interest as a pulp-capping agent for preserving pulp vitality. Hyaluronic acid (HA) is a carbohydrate polymer and a naturally occurring mucopolysaccharide that belongs to the glycosaminoglycan family. It is synthesized on the cytoplasmic surface of plasma membranes and is abundant in humans and other vertebrates. HA plays an essential role in the intercellular adhesion of capillary walls and in the extracellular matrix of connective tissues. The accumulation of HA notably increases during processes such as development, morphogenesis, wound healing, regeneration, malignancy, and inflammation [16–20].

Hyalgan (sodium hyaluronate) is a transparent, colorless, and viscous solution containing a highly purified fraction of hyaluronic acid, derived from rooster combs through a molecular filtration process. The particular fraction of hyaluronic acid used in Hyalgan has a well-defined molecular structure, having an average molecular weight of 500,000–730,000 Dalton (Da). Each 2 ml vial contains 20.0 mg of sodium hyaluronate, 0.1 mg of monobasic sodium phosphate, 17.0 mg of sodium chloride, 1.2 mg of dibasic sodium phosphate and sterile water to make up 2 ml [16].

The materials used for pulp capping must have antibacterial qualities, as bacteria are the main culprits behind dental infections and the failure of pulp therapy [21]. For this investigation, *Enterococcus faecalis*,* Streptococcus mutans*, and *Lactobacillus acidophilus* bacteria were selected for microbiological testing. *E. faecalis* is a facultative anaerobe and frequently found in secondary infections of teeth that underwent endodontic treatment and its principal cause of peri-radicular lesions that appear after endodontic therapy. *E. faecalis* can infiltrate the dentinal tubules and is resistant to high pH [22].

While *Streptcoccus mutans* is the primary pathogen initiating dental caries and frequently present in the oral cavity, *L. acidophilus* is responsible for caries progression as it can ferment carbohydrates into lactic acid and is found in many individuals with active dental decay [22].The agar diffusion method was employed in this in vitro investigation because it is simple to use and is reasonably priced [23]. Because its alkaline pH and calcium-ion release make it an unfavorable habitat for many bacteria, MTA was selected as the control group. The development and metabolism of bacteria may be hampered by their alkalinity and its excellent sealing ability helps prevent microleakage and bacterial penetration, further contributing to its antibacterial effectiveness [24],.

The amniotic membrane possesses antimicrobial properties because of its rich composition of growth factors, anti-inflammatory cytokines, It has anti-fibrotic and antimicrobial properties (including beta-defensins), making it a distinctive treatment for wound care [25]. Hyaluronic acid also has antibacterial properties. It can prevent bacterial growth and inhibit its proliferation. Furthermore, because of its hyper-hydrophilicity and negative charge, which prevent bacterial growth, it has physical characteristics that aid in preventing bacterial adhesion (anti-biofouling effect) [23, 26], Therefore amniotic membrane and hyaluronic acid were selected to test their promising antibacterial effects against some oral pathogens.

The tested materials were incubated for 24 h to fully react and release the active components. Agar plates were incubated at 37 °C under anaerobic conditions to mimic oral temperature and the anaerobic environment in which the selected bacteria grow [24].

### Aim of the study

The purpose of this study was to evaluate the antibacterial effects of amniotic membrane and hyaluronic acid compared with Mineral trioxide aggregate against *Enterococcus faecalis*, *Lactobacillus acidophilus* and *Streptococcus mutans* using an agar diffusion method.

## Materials and methods

### Sample size Estimation

Based on the null hypothesis and using an alpha level of (0.05), a beta of (0.2) i.e. power = 80% and an effect size (f) of (0.682) calculated using the results of a prior study [27]; the predicted sample size (n) was a total of (72) samples (i.e. 24 samples per group). Sample size calculation was performed using G*Power version 3.1.9.7 [28].

A total of 72 Mueller-Hinton agar plates were prepared and inoculated with standardized bacterial suspensions. A completely randomized design was used to assign the plates into three groups (*n* = 24 per group) as follows:

- Group I: MTA (positive control group).
- Group II: Dehydrated amniotic membrane (study group).
- Group III: Hyaluronic acid (study group).

### Ethical approval

The study was reviewed and approved by the research ethics committee of the Faculty of Dentistry, Ain Shams University with reference number (FDASU-Rec ID022205). All procedures were carried out in accordance with the ethical principles outlined in the Declaration of Helsinki. The study was carried out over a period of six months, from January 2024 to June 2024.

### Study setting

The study was carried out at the Department of Microbiology, Ain Shams University, Faculty of Medicine.

### Study design

 This study is an invitro study.

#### Study procedure

The antibacterial efficacy of MTA (Neo-Putty, Avalon Biomed, Houston, TX, USA), amniotic membrane (Vera Graft, San Antonio, USA) and hyaluronic acid (Hyalgan; high molecular weight 500,000–730,000 Dalton (Da), 20 mg/2 mL: Fidia Pharmaceutical, Italy) were assessed using the agar diffusion technique [27].The agar diffusion assays were performed at the Faculty of Science, Ain Shams University, Egypt using the bacterial strains *Enterococcus faecalis* (ATCC 29212),*Streptococcus mutans* (ATCC 25175) and *L. acidophilus*(ATCC 4356). The microorganisms were sourced from culture collections maintained by the Faculties of Science and Agriculture at Ain Shams University [28].

*E. faecalis and S. mutans* were cultured anaerobically in Brain–Heart Infusion (BHI) broth, whereas *L. acidophilus* was cultured in Man- Rogosa-Sharpe (MRS) broth. Bacterial cultures were activated and incubated in a shaker incubator (Heraeus, Germany) at 37 °C for 24 to 48 h [27, 29].Following incubation, bacterial dilutions were prepared to achieve a concentration of 5 × 10^8^ using 0.5 McFarland turbidity [27, 30]. *E. faecalis* and *S. mutans* strains were inoculated onto the surfaces of solidified BHI agar plates, while L. acidophilus was spread on MRS broth solidified agar plates using sterile swabs [29, 30].

For the microbiological evaluation, Brain Heart Infusion (BHI) agar and De Man, Rogosa, and Sharpe (MRS) agar were prepared according to the manufacturer’s instructions. BHI agar was used for culturing Enterococcus faecalis and Streptococcus mutans, while MRS agar was used for Lactobacillus acidophilus. The appropriate amount of agar powder (52 g/L for BHI and 62 g/L for MRS) was weighed and dissolved in distilled water with continuous stirring. The mixtures were then brought to a gentle boil to ensure complete dissolution. Following this, the media were sterilized in an autoclave at 121 °C for 15 min. After autoclaving, the agar solutions were allowed to cool to approximately 45–50 °C before being poured into sterile Petri dishes under aseptic conditions. The plates were left to solidify at room temperature and subsequently stored at 4 °C until inoculation [27, 29].

#### Agar diffusion test

A total of seventy-two plates were arranged and then randomly allocated into three groups (*n* = 24 for each material). Two wells (4 mm height x 5 mm diameter) were created in each agar plate for testing Mineral trioxide aggregate and hyaluronic acid, while the amniotic membrane was directly placed onto the agar surface [30]. The tested materials were applied as follows: Group I: 1 ml of Neoputty MTA was injected directly into the wells. Group II: Dehydrated amniotic membrane was cut into 4 mm x 4 mm pieces using sterile scissors, hydrated in saline for 1 min, and placed directly on the agar surface. Group III: Each well was filled with 1 ml HA. To ensure anaerobic conditions, the plates were stored in a sealed jar with a candle and incubated at 37 °C for 24 h [28, 29].Once the incubation period was complete, the inhibition zones around each test material were measured using a digital caliper (Mitutoya, Japan) [27].

### Statistical analysis

The numerical data were presented as the mean and standard deviation, the homogeneity of variances and normality were assessed by examining the data distribution and performing Shapiro-Wilk and Levene tests, respectively. The data followed a normal distribution, though the assumption of homogeneity was not met. Therefore, one-way ANOVA was used for analysis, followed by the Games-Howell post hoc test. A significance level of *p* < 0.05 was applied. Statistical analysis was conducted using R software version 4.3.2 for Windows [21].

## Results

### Intergroup comparison of bacterial Inhibition zones (mm)

**Fig. 1:**
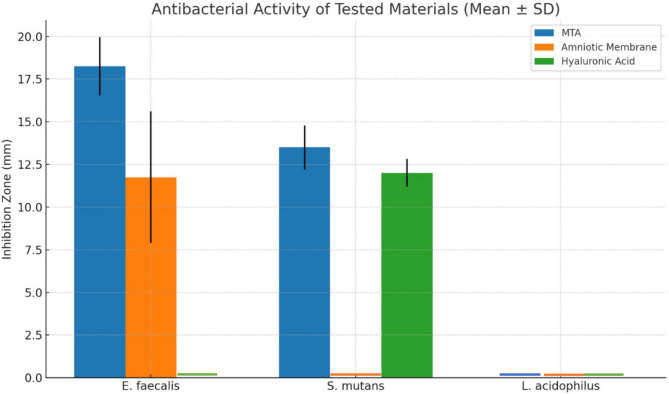
Bar chart illustrating the mean inhibition zone diameters (mm) for MTA, amniotic membrane, and hyaluronic acid against *E. faecalis* and *S. mutans*. None of the tested materials exhibited antibacterial activity against *L. acidophilus.* Images of representative agar diffusion plates for each bacterial strain are also shown in figure (2,3,4 and 5).

The intergroup comparison of bacterial inhibition zones (mm) for *E. faecalis*, *S. mutans*, and *L. acidophilus* is summarized in Table (1.) and Figure (1). A statistically significant difference was observed among the groups for *E. faecalis* and *S. mutans* (*p* < 0.001), whereas no significant inhibition was observed for *L. acidophilus* (*p* = NA).

For *E. faecalis*, MTA exhibited the highest antibacterial activity (18.25 ± 1.71 mm), followed by the amniotic membrane (11.75 ± 3.86 mm), with no significant difference between them (*p* > 0.05). Hyaluronic acid showed no antibacterial effect (0.00 ± 0.00 mm). Post hoc comparisons revealed that both MTA and amniotic membrane had significantly greater effects than hyaluronic acid (*p* < 0.001).

For *S. mutans*, MTA again showed the highest inhibition (13.50 ± 1.29 mm), followed closely by hyaluronic acid (12.00 ± 0.82 mm), with no significant difference between these two groups (*p* > 0.05). However, the amniotic membrane showed no inhibitory effect (0.00 ± 0.00 mm), and both MTA and hyaluronic acid were significantly more effective than the amniotic membrane (*p* < 0.001).

Regarding *L. acidophilus*, none of the tested materials exhibited any antibacterial activity, and there were no statistically significant differences among the groups (*p* = NA).

**Table 1.** Intergroup comparison of mean ± SD values of bacterial inhibition zones (mm) for all tested bacteria

Bacteria	MTA (Group I)	Amniotic Membrane (Group II)	Hyaluronic Acid (Group III)	*p*-value
*E. faecalis*	18.25 ± 1.71ᴬ	11.75 ± 3.86ᴬ	0.00 ± 0.00ᴮ	< 0.001*
*S. mutans*	13.50 ± 1.29ᴬ	0.00 ± 0.00ᴮ	12.00 ± 0.82ᴬ	< 0.001*
*L. acidophilus*	0.00 ± 0.00	0.00 ± 0.00	0.00 ± 0.00	NA

*Means with different superscript letters within the same row are significantly different (*p* < 0.05)

*; statistically significant

**Fig. 2:**
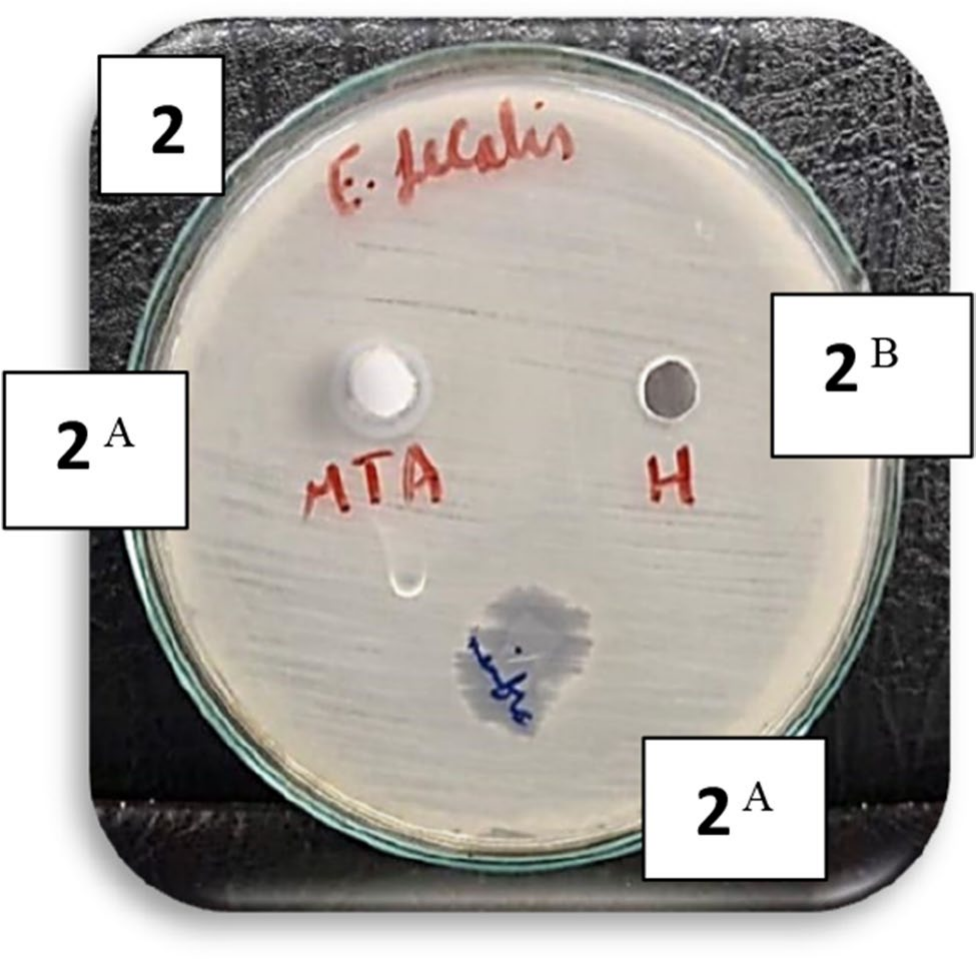
Agar diffusion plate with zones of inhibition against *E. Faecalis* in different groups. (Different superscript indicate significant difference)

**Fig. 3:**
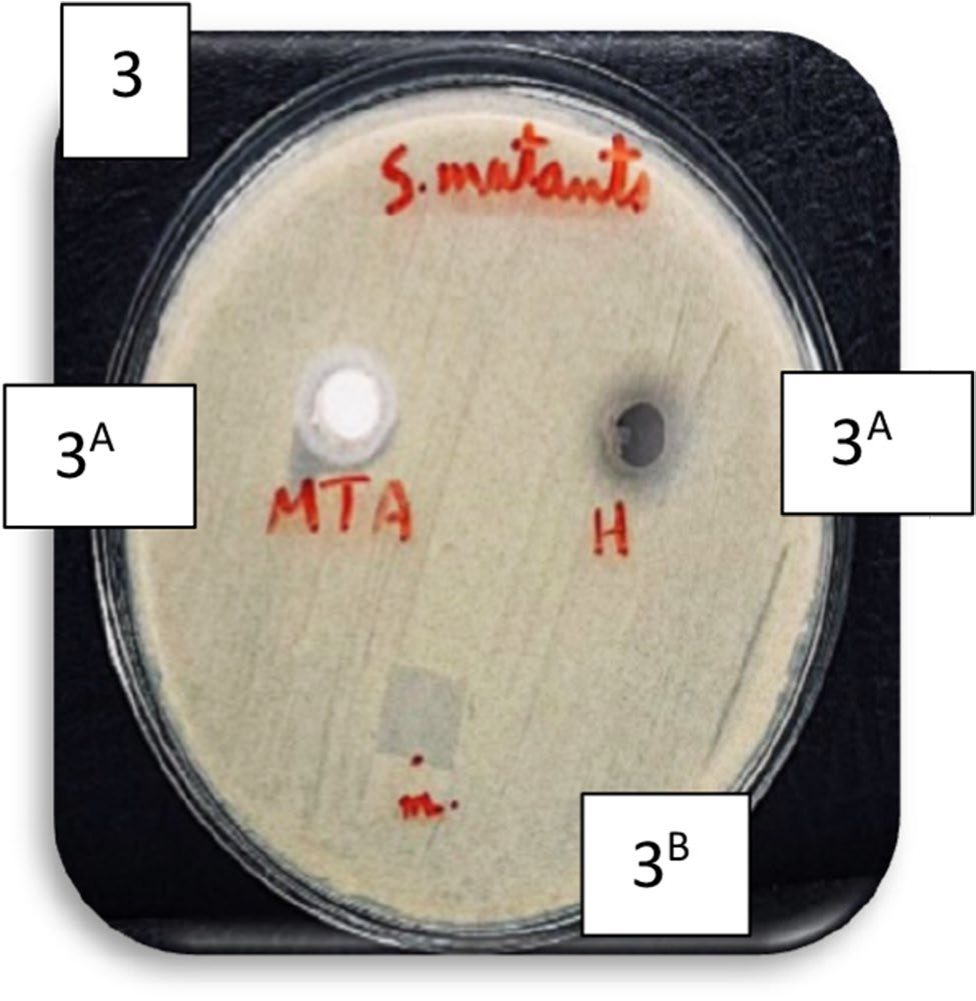
Agar diffusion plate with zones of inhibition against *S.mutans* in different groups. (Different superscript indicate significant difference)

**Fig. 4:**
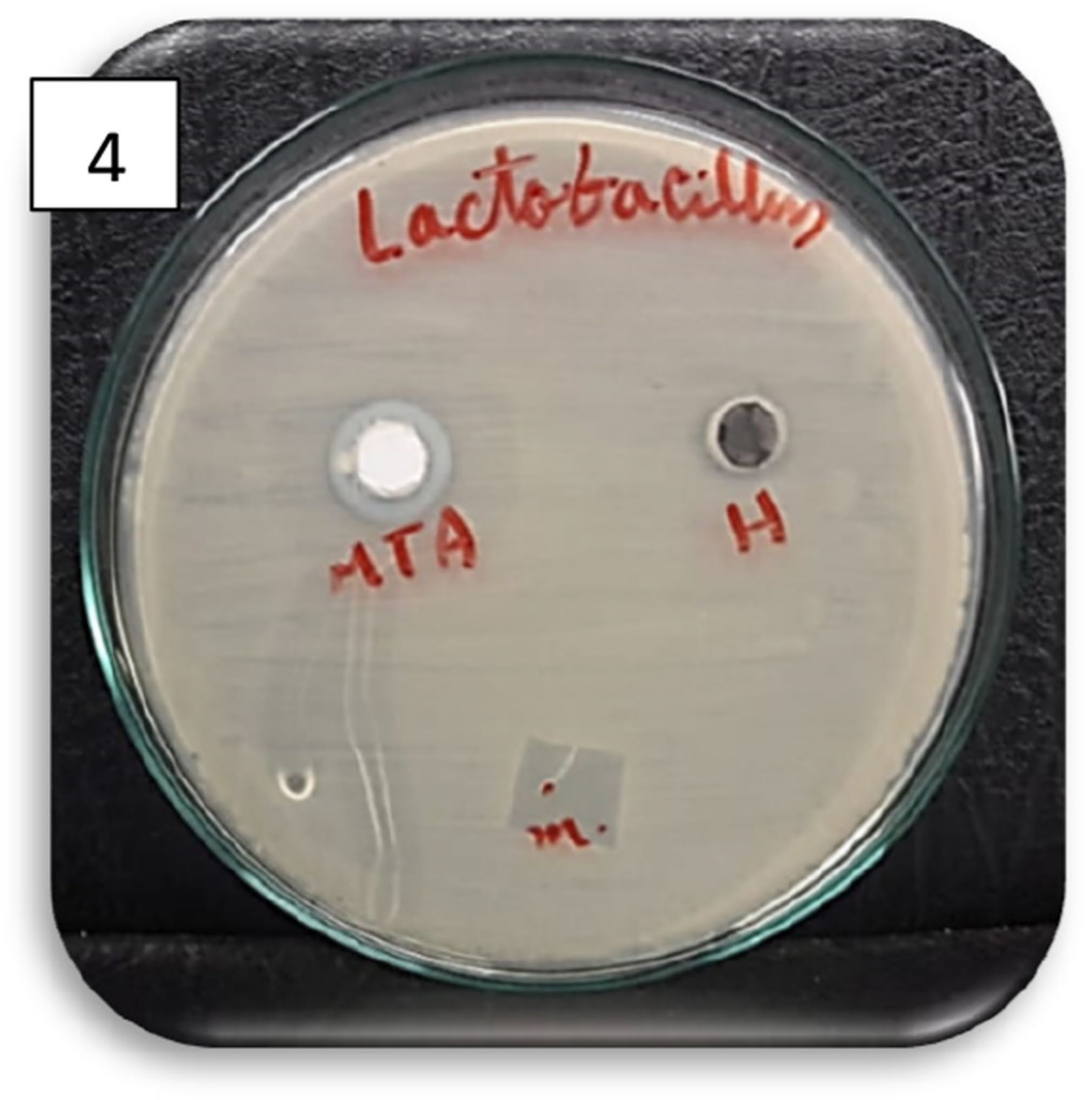
Agar diffusion plate with zones of inhibition against *L.acidophilus* in different groups. (None of the tested materials showed any antibacterial effect against *L.acidophilus*, with no statistically significant differences among groups (*p*: NA)

**Fig. 5:**
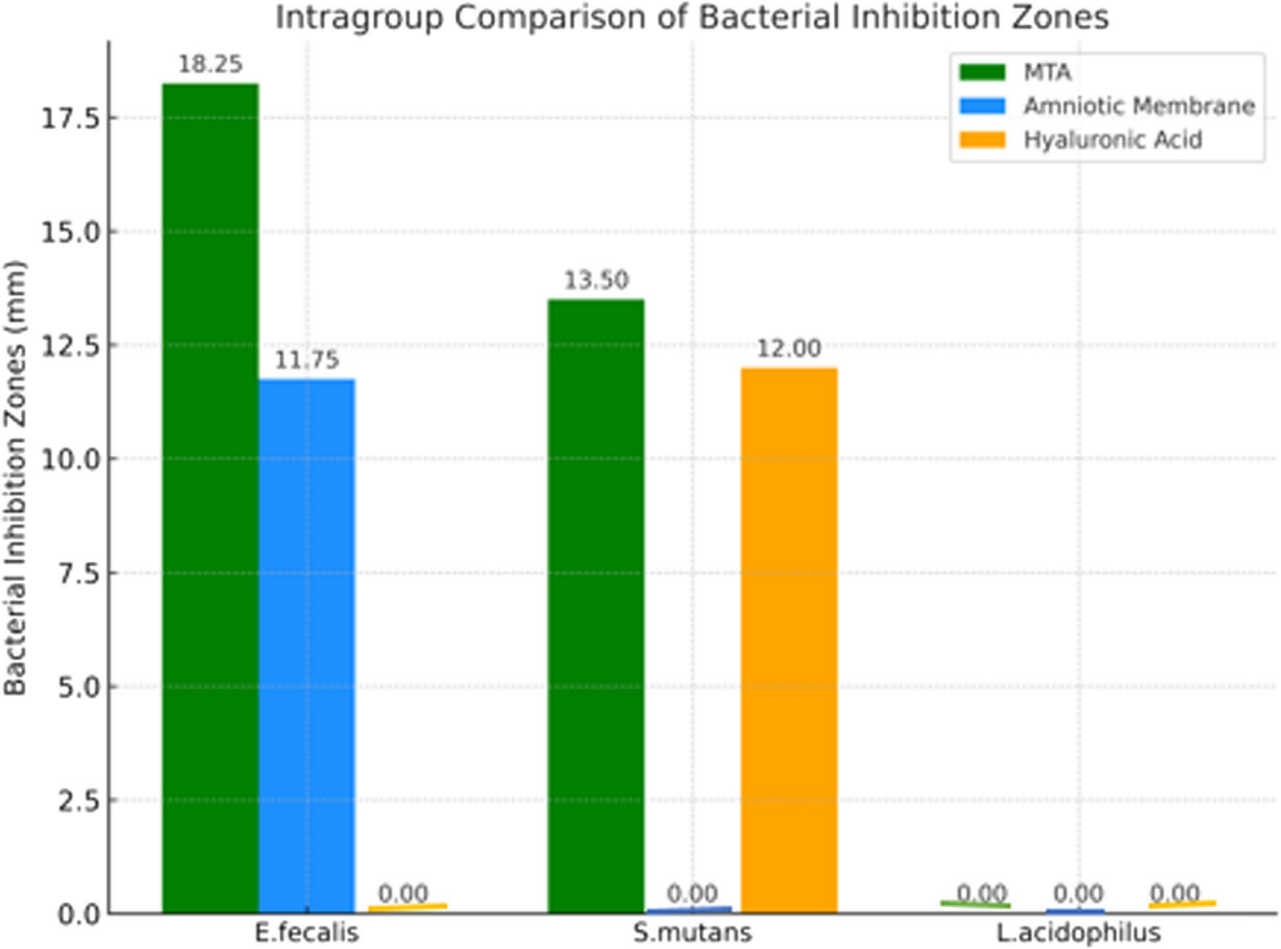
Bar chart represents the mean inhibition zones for each bacterial strain within each group (MTA, Amniotic Membrane and Hyaluronic Acid)

### Intragroup comparison of bacterial Inhibition zones (mm)

Intragroup comparison of bacterial inhibition zones (mm) for *E. faecalis*,* S. mutans*,* and L. acidophilus* within each tested group (MTA, Amniotic Membrane, and Hyaluronic Acid). The results show that MTA exhibited significant antibacterial activity against both *E. faecalis and S. mutans*, while Amniotic Membrane showed activity only against *E. faecalis*. Hyaluronic Acid was effective only against *S. mutans*. None of the tested materials showed antibacterial effect against *L. acidophilus*. Statistically significant differences (*p* < 0.001) were observed within each group, as indicated by different superscript letters (Table 2.).

**Table 2.** Intragroup Comparison of mean ± SD values of Bacterial Inhibition Zones (mm) within each group

Bacteria	MTA (GroupI)	Amniotic Membrane (Group II)	Hyaluronic Acid (Group III)	*p*-value
*E. faecalis*	18.25 ± 1.71ᴬ	11.75 ± 3.86ᴬ	0.00 ± 0.00ᴮ	< 0.001*
*S. mutans*	13.50 ± 1.29ᴮ	0.00 ± 0.00ᴮ	12.00 ± 0.82ᴬ	< 0.001*
*L. acidophilus*	0.00 ± 0.00ᶜ	0.00 ± 0.00ᴮ	0.00 ± 0.00ᴮ	< 0.001*

*Means with different superscript letters are significantly different (*p* < 0.05)

## Discussion

This investigation is one of the first to evaluate and compare the antimicrobial activity of Hyaluronic Acid (HA) and Amniotic Membrane (AM) with Mineral Trioxide Aggregate (MTA) against three significant oral pathogens: *Enterococcus faecalis*,* Streptococcus mutans*, and *Lactobacillus acidophilus*. It presents new insights into the antibacterial potential of biologically derived materials for pulp capping, an area that remains relatively underexplored in paediatric dental research.

The study also holds several strengths, it utilized clinical-grade formulations of HA and AM, enhancing the clinical relevance of the findings. The experimental design adhered to standardized protocols for material preparation, bacterial culture, and agar diffusion testing, ensuring the reproducibility and reliability of results. Furthermore, by targeting three key bacterial species associated with pulp infections, the study provided a comprehensive assessment of antimicrobial effectiveness across multiple microbial threats commonly encountered in paediatric endodontic.

Results of present study showed that hyaluronic acid had no effect on *E. faecalis*, but MTA and amniotic membrane demonstrated antibacterial activity against the bacteria with no significant difference between them.

This may be due to the presence of tricalcium and di-calcium silicate, which form an alkaline calcium silicate gel and release hydroxyl ions. This alkalinity creates an unfavorable environment for microbial growth [30]. These findings are consistent with Ravindran et al. [31] who demonstrated that MTA had antibacterial effects against *E. faecalis*. In contrast, Kim et al. [32] revealed that *E. faecalis* exhibited the highest resistance to both MTA Angelus and ProRoot MTA in disc diffusion assays.

Regarding group II (amniotic membrane), the results agreed with Niestrata et al. [25] who reported an antibacterial effect of amniotic membrane against *E. faecalis* and emphasized the therapeutic potential of the amniotic membrane in improving the healing process of keratitis caused by *E.* faecalis. This can be explained by its ability to promote regeneration and inhibit bacterial growth.

However, the current findings conflict with those of Heidarzadeh et al. [33] that revealed no antibacterial effect of amniotic membrane against *E. faecalis*. The observed results may be due to variations in the methodologies used. The authors used a smaller piece of amniotic membrane (1.5 mm×1.5 mm) than used in the current study (4 × 4 mm).

In group III, there was complete absence of an inhibition zone of hyaluronic acid against *E. faecalis*. This result was in agreement with the findings of Bustamante et al. [34] who reported that HA had no antibacterial effect against *E. faecalis.* This may be because hyaluronic acid is dose and concentration dependant and *E.faecalis* is a highly resistant bacteria.

According to Ardizzoni et al. [35], hyaluronic acid demonstrated inhibitory effects against *Enterococcus faecalis*, a finding that disagrees with our finding, the difference could be attributed to higher concentration (4 mg/ml) and more diluted bacterial load (5 × 10^3^) than used in the current study.

This study showed that MTA and hyaluronic acid had significant antibacterial effects against S. *mutans*. Unlike other materials, the amniotic membrane showed no effectiveness against *S. mutans*. These findings align with those of Ravindan et al. ^[31]^ and Sipert et al. [36].

Shetty et al. [37] also indicated that the amniotic membrane exhibited no antibacterial effect against *S. mutans*. In contrast, Ramasamy et al. [38] indicated that zinc oxide nanoparticles derived from human amniotic membrane proteins (HAMP-ZnO NP) had great inhibitory effects against *S. mutans*. This may be due to the synergistic antibacterial action of the ZnO nanoparticles and amniotic membrane proteins.

The inability of AMs in Group II to exhibit antibacterial effects against S. mutans may be due to variations in the concentrations of antimicrobial peptides among different types of amniotic membranes. This variation may be caused by several factors, such as gestational age, processing and preservation methods, donor variability, genetic differences, inflammatory and storage conditions [39–43].

The results of group III (HA) agreed with those of Tüzüner et al. [23] who observed that HA demonstrated antibacterial effects against *S. mutans* using the agar diffusion test. This is may be due to the ability of hyaluronic acid to inhibit bacterial HA lyase synthesis, preventing the proliferation of *S. mutans* in HA coatings. This phenomenon is referred to as the antibiofouling property of HA [44]. Pirnazar et al. [26] also observed that HA effectively combats *S. mutans* when using high molecular weight HA, whereas low molecular weight HA exhibited no antibacterial effect. This finding suggests that *S. mutans* takes longer time to break longer polymeric chains of high molecular-weight HA.

The results revealed that *L. acidophilus* was resistant to all tested materials *(MTA*, Amniotic membrane and Hyaluronic acid*).T*ested materials may be biologically inert with no chemical reactions or toxic substances that could suppress the growth of *L. acidophilus*, which allowed the bacterium to persist in its growth without being affected.

In contrast, Ünlü et al. [45] indicated the significant antibacterial activity of MTA against *L. acidophilus*. This discrepancy may be due to the use of the broth microdilution method, which is more sensitive than the agar diffusion method. Additionally, Ehsani et al. [46] demonstrated the antibacterial effect of MTA Fillapex sealer on *Lactobacillus*, which may be attributed to the difference in composition and physical form between MTA Fillapex and NeoPutty MTA. Furthermore, Bakır et al. [47] found that MTA Angelus exhibited strong antibacterial activity against *L. acidophilus* strains when tested using a bacterial load of 1.5 × 10⁸ CFU/ml. This bacterial concentration may have influenced the observed antibacterial effects.

Moreover, Shetty et al. [37] reported that amniotic membrane had antibacterial effect against L.acidophilus. This may be due to the use of fresh amniotic membranes, which retain their native structure biological components, and antimicrobial proteins, whereas the present study used dehydrated amniotic membranes in which some antimicrobial peptides (e.g., defensins, lysozymes, and lactoferrin) may have been degraded or inactivated, leading to reduced antibacterial effects.

Therefore, this study rejected the null hypothesis, confirming a significant variation in the antibacterial effects of the materials evaluated. MTA and the amniotic membrane exhibited greater antibacterial activity against *E. faecalis* compared with hyaluronic acid. In contrast, both MTA and hyaluronic acid exhibited a more pronounced antibacterial effect against *S. mutans* compared to the amniotic membrane. Nevertheless, none of the three materials tested showed antibacterial activity against *L. acidophilus. The* current study evaluated the antibacterial effect of two emerging materials in pulp therapy (Amniotic membrane, Hyaluronic acid)compared to MTA as the gold standard against common oral pathogens.

However, the study had some limitations, including difficulties in handling and cutting the amniotic membrane due to its fragility. The tested materials were directly applied to the bacteria, which did not fully mimic the clinical environment in which bacteria are present within the dentinal tubules. The high expense of the amniotic membrane represents an additional limitation. To enhance the accuracy and reliability of antimicrobial evaluations, future studies are recommended to incorporate broth microdilution or time-kill assays. These methods offer greater sensitivity and quantitative assessment compared to agar diffusion, particularly when testing viscous or slow-diffusing materials such as MTA, amniotic membrane, and hyaluronic acid and suggesting further in vivo and histological studies to support the clinical application of HA and AM.

## Conclusion

MTA was superior in all effective comparisons against *E. faecalis* and *S. mutans*, whereas amniotic membranes demonstrated strong antibacterial effects against only *E. faecalis*. Hyaluronic acid exerted strong antibacterial effects against *only S. mutans.* None of the evaluated materials demonstrated any inhibitory effect on *L. acidophilus*.

## Supplementary Information

Supplementary Material 1.

## Data Availability

No datasets were generated or analysed during the current study.
