# Supplementary material for: Comparative antibacterial efficacy of MTA, amniotic membrane, and hyaluronic acid in pulp therapy for primary teeth
Source: BMC Oral Health. 2025 Nov 5;25:1747. doi: 10.1186/s12903-025-07038-3 (PMC12587748; doi:10.1186/s12903-025-07038-3)
Supplement: Supplementary file 1 — Supplementary Material 1. [file 12903_2025_7038_MOESM1_ESM.docx]

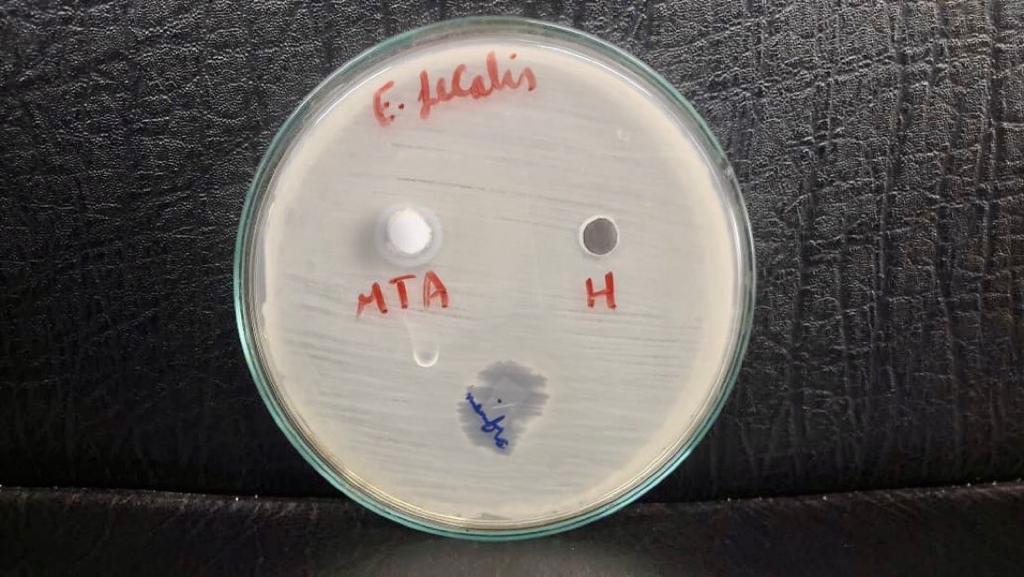


**Figure (1):** Agar diffusion plate with zones of inhibition against *E. Faecalis*in different groups.


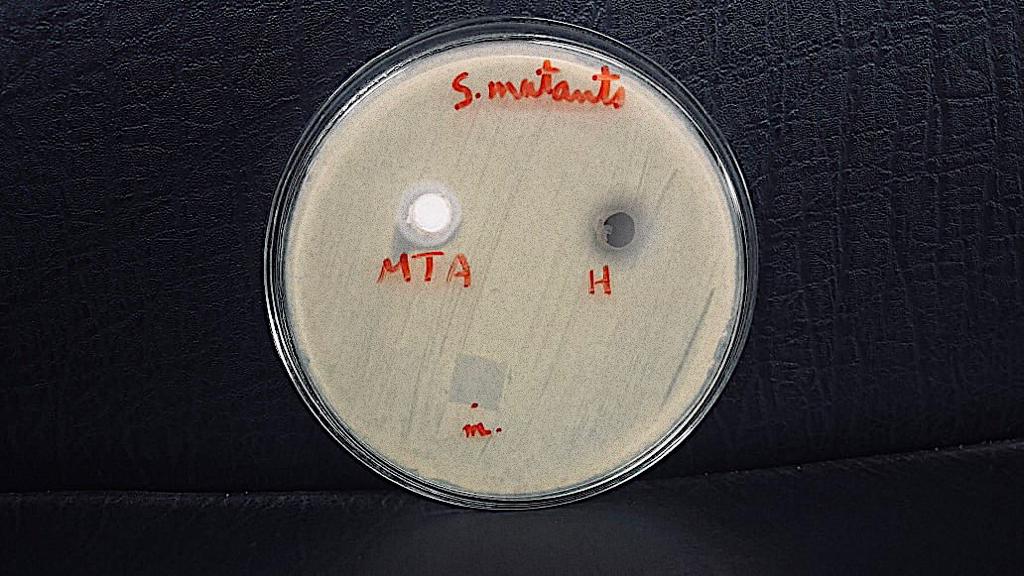


**Figure(2):** Agar diffusion plate with zones of inhibition against *S.mutans* in different groups.


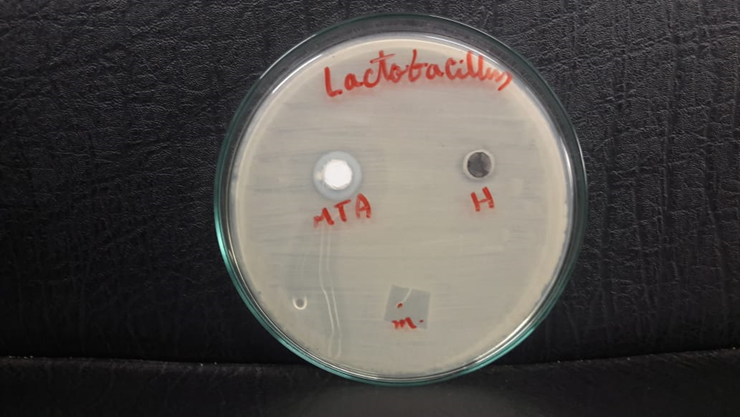


**Figure (3):** Agar diffusion plate with zones of inhibition against L.acidophilus in different groups.


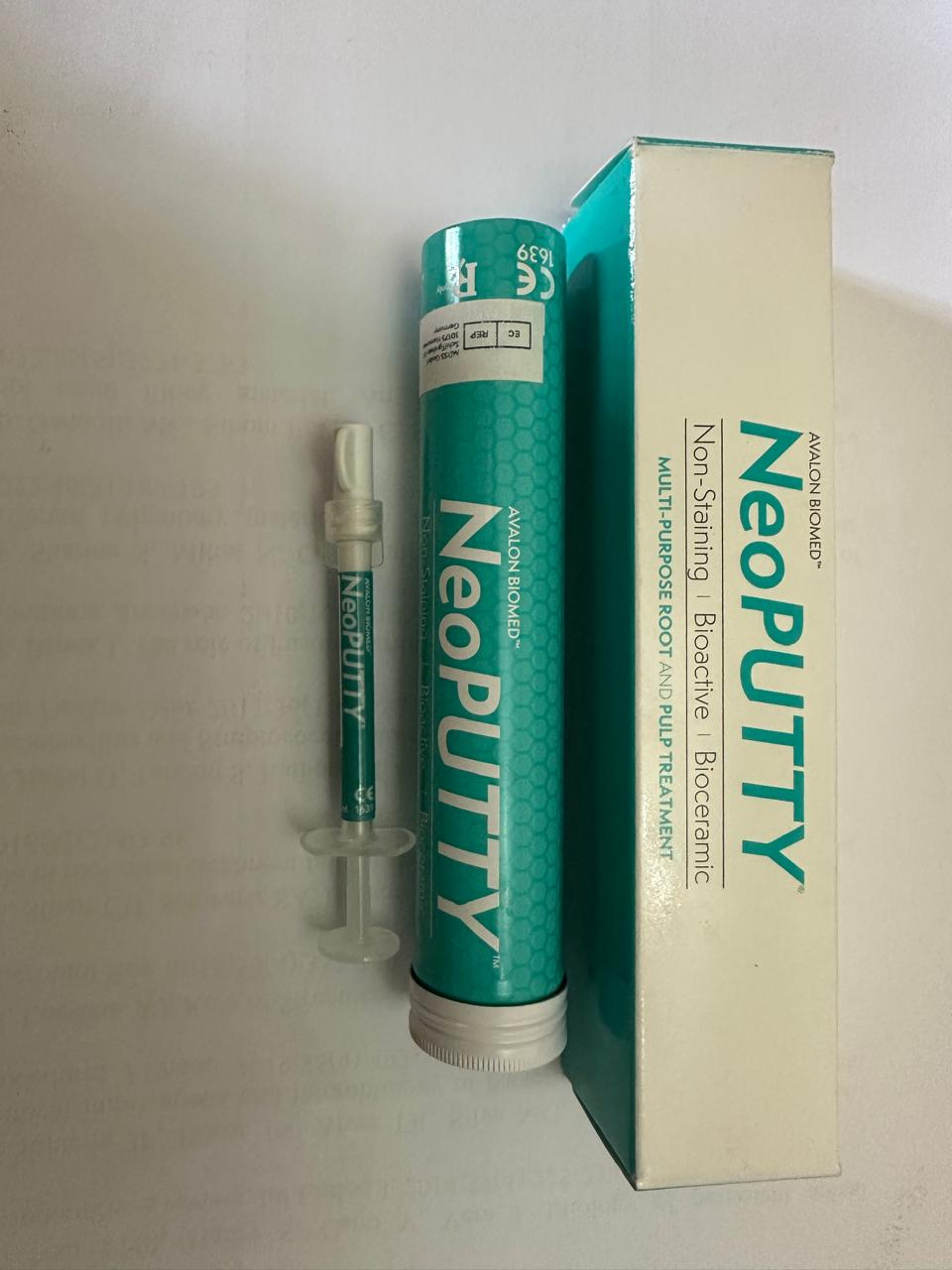


**Figure (4):** Mineral Trioxide aggregate (Group I)


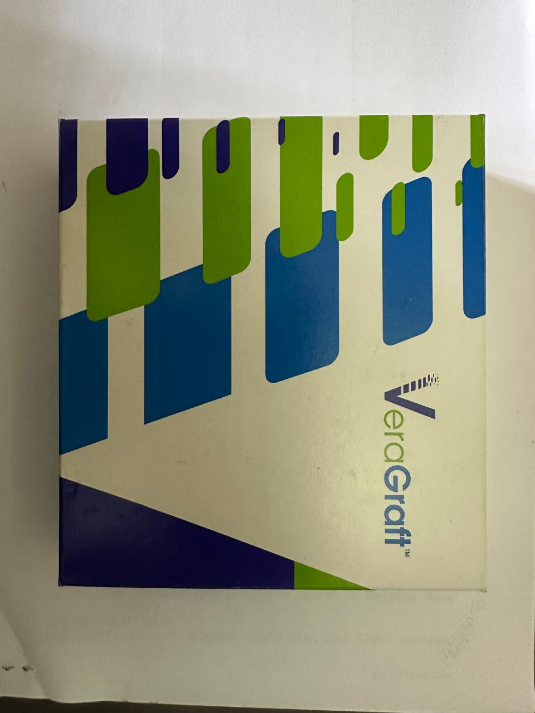

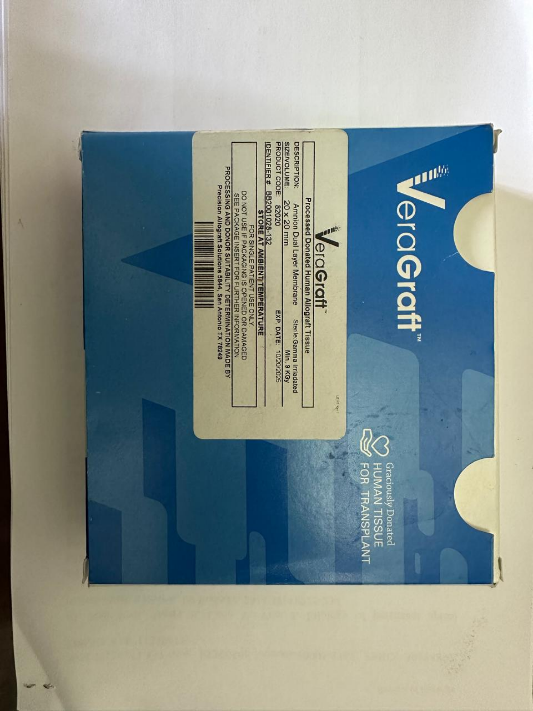


5B

5A


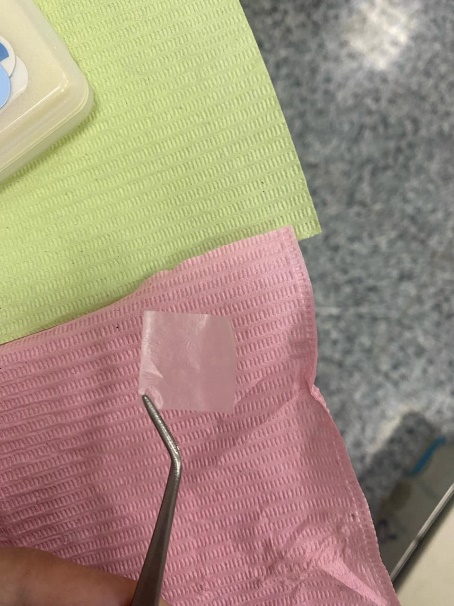


5C

**Figure (5A,5B,5C):**Amniotic Membrane group II


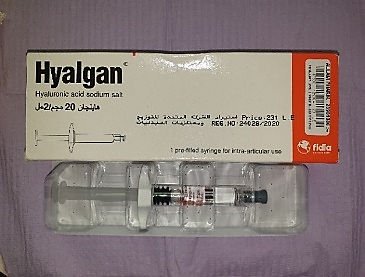


**Figure (6):** Hyaluronic acid group III
